# The role of adjuvant radiotherapy in patients with malignant phyllodes tumor of the breast: a propensity-score matching analysis

**DOI:** 10.1007/s12282-020-01135-7

**Published:** 2020-08-03

**Authors:** Wen Zhao, Qi Tian, Andi Zhao, Biyuan Wang, Jiao Yang, Le Wang, Lingxiao Zhang, Danfeng Dong, Ling Chen, Jin Yang

**Affiliations:** Department of Medical Oncology, The First Affiliated Hospital of Xi’an Jiaotong University, No 277 Yanta West Road, Xi’an, 710061 Shaanxi People’s Republic of China

**Keywords:** MPTB, SEER, Adjuvant radiotherapy, PSM, Survival

## Abstract

**Background and objectives:**

Malignant phyllodes tumor of the breast (MPTB) is a kind of rare tumor. Our objective was to investigate the role of adjuvant radiotherapy (RT) in MPTB patients.

**Methods:**

MPTB patients were identified in the Surveillance, Epidemiology and End Results (SEER) database. Kaplan–Meier curves and multivariable Cox proportional hazards analyses were conducted to determine the effect of adjuvant RT on MPTB patients. Propensity-score matching (PSM) method was used to balance the clinicopathological characteristics.

**Results:**

A total of 1353 MPTB patients were included in our study and the median follow-up time was 99 months (range: 0–331 months). 16.7% (226) MPTB patients received adjuvant RT, of which 49.1% (111) received mastectomy and 50.9% (115) underwent breast conservation surgery (BCS). Patients receiving adjuvant RT were more likely to be white, with better differentiation and larger tumors (*p* < 0.05). Multivariate analysis showed that poorer tumor differentiation grade, larger tumor size, and lymph node metastasis were associated with reduced survival while BCS was a protective factor of disease-specific survival (DSS) (HR 0.297; 95% CI 0.184–0.480) and overall survival (OS) (HR 0.445; 95% CI 0.321–0.616). After PSM, survival curves showed patients did not achieve an improved OS or DSS from adjuvant RT (*p* > 0.05). In subgroup analysis, no subgroup benefited from adjuvant RT. Exploratory analysis showed a survival benefit trend from adjuvant RT in patients with tumor larger than 50 mm and undergoing BCS.

**Conclusions:**

Among MPTB patients, adjuvant RT did not improve OS or DSS. In patients with tumor larger than 50 mm and receiving BCS, a survival benefit trend from adjuvant RT existed.

**Electronic supplementary material:**

The online version of this article (10.1007/s12282-020-01135-7) contains supplementary material, which is available to authorized users.

## Introduction

Phyllodes tumor of the breast (PTB) is rare, of which the incidence rate is 2% to 3% in all breast fibrous epithelial tumors, or 0.3% to 1.0% in all breast tumors [1, 2]. It was in 1838 that Johannes Muller first reported this tumor and named it as cystosarcoma phyllodes, because of its huge neoplasia with a cystic lobulated section. Until 1981, the international histological classification group of World Health Organization (WHO) renamed it as phyllodes tumor and divided it into three subtypes: benign, borderline and malignant, according to pathological features [3, 4].

Malignant phyllodes tumor of breast (MPTB) comprises 20% of all PTB [2] and is characterized by the aggression of clinical features and propensity for local recurrence and distant metastasis [5]. It occurs most frequently in women of child-bearing stage [6] and some patients have a history of benign breast diseases, including fibroadenoma [2]. They usually present with an insidious onset and slow progression, but rapid growth in a short term. The lesions are often unilateral, single, nodular, painless masse with size varying widely, ranging from smaller than 1 cm to 40 cm [7]. As MPTB performs an unpredictable and sometimes aggressive neoplasm, it matters a lot to investigate the features of clinicopathological factors and their prognostic roles.

Surgery is the preferred treatment for MPTB and lymph nodes resection is not recommended, as lymph node metastases are rare. The local recurrence rate is high in PTB patients, especially in MPTB patients, up to 40% of all histological types [5, 8–10]. However, the efficacy of adjuvant radiotherapy (RT) is not clear. Many investigators have focused on this topic, ending with paradoxical results [9–14]. Besides, many studies analyzed subtypes of PTB altogether or borderline and malignant PTB combined, included only a minority of malignant tumors and generally evaluated local and distant disease recurrence rate without a report on disease-specific survival (DSS) or overall survival (OS). Importantly, the imbalance in the studied population led to the interpretation of the benefit of adjuvant RT questionable.

The aim of this study was to investigate whether MPTB patients benefit from adjuvant RT through an analysis of a large cohort of well-characterized patients, identified from the Surveillance, Epidemiology and End Results (SEER) database.

## Materials and methods

### Patients

We obtained data from the National Cancer Institute’s SEER program, which consists of 18 population-based cancer registries, between 1988 and 2015. SEER is an open-access resource for cancer-based demographic and clinical information, as well as treatment and patient survival. SEER*Stat Version 8.3.5 (http://www.seer.cancer.gov/seerstat) was used to identify eligible patients.

The inclusion criteria of MPTB patients were listed as follows: female, year of diagnosis from 1988 to 2015, breast tumor as the first and only malignant tumor diagnosis, pathologically confirmed MPTB (ICD-O-3 9020/3), unilateral tumor, surgical treatment with either mastectomy or BCS and receiving adjuvant RT or not. Demographic variables included age at diagnosis (≤ 35, 35-55, and > 55 years), and race (white, black, and others). Tumor characteristics included laterality (left and right), T stage (T0, T1, T2, T3, and T4), N stage (N0, N1, N2, and N3), and grade (well differentiated (I), moderately differentiated (II), poorly differentiated (III) and undifferentiated (IV)). Therapies included RT, and surgery of the primary tumor.

### Propensity-score matching (PSM)

Because it was a nonrandomized and retrospective analysis, unbalanced baseline characteristics may lead to selection bias and influence the decision to administer RT. The propensity score was defined here as the probability of being assigned to surgery plus adjuvant RT cohort or surgery alone cohort was given the clinicopathological characteristics. It was estimated using the logistic regression model that had been established from the factors potentially affecting a decision of treatment modalities. These factors included age at diagnosis, race, laterality, differentiation grade, tumor size, lymph node metastasis and surgery procedure. Patients who received adjuvant RT were matched to other patients based on the calculated scores with an algorithm of the nearest neighbor 1:1 matching [15].

### Statistical analysis

Patient demographics, tumor characteristics, and surgery procedure were compared between those who received adjuvant RT and those who did not using the Chi square test. Overall survival (OS) was used as the primary study outcome and was defined as the time from tumor diagnosis to death from any cause. Disease-specific survival (DSS) was also compared, defined as the time from tumor diagnosis to death due to MPTB. Kaplan–Meier method was used to generate survival curves and multivariate Cox proportional hazard models to identify the prognostic factors associated with DSS and OS. Hazard ratios (HRs) and 95% confidence intervals (95% CIs) were reported. All analyses were performed with SPSS (version 24.0; SPSS, Inc., Chicago, IL). Statistical significance was assumed at *p* values < 0.05.

## Results

1353 MPTB patients were included in our study and the median follow-up time was 99 months (range 0–331 months). 226 (16.7%) received adjuvant RT, while 1127(83.3%) patients did not. 51.6% (698) were diagnosed between 35 and 55 years and the mean age at diagnosis was 46 years (range 9–96 years). Among MPTB patients with known grade information, 47.6 percent were diagnosed with a poorly differentiated or undifferentiated tumor. 43.9% MPTB patients were diagnosed with tumor larger than 50 mm and the median tumor size was 46 mm (range 3–360 mm). Only 1.3% of patients had lymph nodes metastasis. 564 (41.7%) underwent mastectomy and 789 (58.3%) underwent BCS. When compared with patients in the surgery alone cohort, those in the surgery plus adjuvant RT cohort were more likely to be white, with better-differentiated tumor and larger size tumor, as well as receiving mastectomy. Demographics, tumor characteristics, and therapy information of MPTB patients were shown in Table 1.

**Table 1:** The clinicopathological characteristics of MPTB patients according to the condition of adjuvant RT before PSM and after PSM

Variable	All patients	Initial cohort			PSM cohort		
	Number (%)	Surgery alone	Surgery + RT	*P* value	Surgery alone	Surgery + RT	*P* value
		Number (%)	Number (%)		Number (%)	Number (%)	
Age (years)				0.450			0.925
≤ 35	177 (13.1)	151 (13.4)	26 (11.5)		27 (12.2)	26 (11.5)	
35–55	698 (51.6)	573 (50.8)	125 (55.3)		125 (56.3)	125 (55.3)	
> 55	478 (35.3)	403 (35.8)	75 (33.2)		70 (31.5)	75 (33.2)	
Race				0.030			0.114
White	995 (73.5)	813 (72.1)	182 (80.5)		192 (86.5)	182 (80.5)	
Black	150 (11.1)	130 (11.5)	20 (8.8)		18 (8.1)	20 (8.8)	
Others and unknown	208 (15.4)	184 (16.3)	24 (10.6)		12 (5.4)	24 (10.6)	
Laterality				0.047			0.942
Left	655 (48.4)	532 (47.2)	123 (54.4)		120 (54.1)	123 (54.4)	
Right	698 (51.6)	595 (52.8)	103 (45.6)		102 (45.9)	106 (45.6)	
Grade				< 0.001			0.948
I	162 (12.0)	118 (10.5)	44 (19.5)		42 (18.5)	44 (19.5)	
II	199 (14.7)	165 (14.6)	34 (15.0)		30 (13.5)	34 (15.0)	
III + IV	328 (24.2)	291 (25.9)	37 (16.4)		37 (16.7)	37 (16.4)	
Unknown	664 (49.1)	553 (49.1)	111 (49.1)		114 (51.4)	111 (49.1)	
Tumor size(mm)				< 0.001			0.416
≤ 50	759 (56.1)	668 (59.3)	91 (40.3)		99 (44.6)	91 (40.3)	
50–100	364 (26.9)	294 (26.1)	70 (31.0)		71 (32.0)	70 (31.0)	
> 100	230 (17.0)	165 (14.6)	65 (28.8)		52 (23.4)	65 (28.8)	
Lymph node				0.935			0.248
Negative	1336 (98.7)	1113 (98.8)	223 (98.7)		0 (0.0)	3 (1.3)	
Positive	17 (1.3)	14 (1.2)	3 (1.3)		226 (100.0)	223 (98.7)	
Surgery procedure				0.013			0.388
Mastectomy	564 (41.7)	453 (40.2)	111 (49.1)		100 (45.0)	111 (49.1)	
BCS	789 (58.3)	674 (59.8)	115 (50.9)		122 (55.0)	115 (50.9)	

*RT* radiotherapy, *MPTB* malignant phyllodes tumor of the breast, *PSM* propensity-score matching, *Grade* I well differentiated; II moderately differentiated; III poorly differentiated; IV undifferentiated, *BCS* breast conservation surgery

We identified the prognostic factors of MPTB patients. Poorer tumor differentiation grade, larger tumor size, and lymph node metastasis were associated with reduced DSS, and the latter two were negatively associated with OS in both univariate and multivariate Cox proportional hazard models (Table 2) while BCS was a protective factor of DSS (HR 0.297; 95% CI 0.184–0.480) and OS (HR 0.445; 95% CI 0.321–0.616). However, we didn’t find the protective role of adjuvant RT in MPTB patients. The detailed information on risk factors was listed in Table 2.

**Table 2:** Prognostic factors for DSS and OS in MPTB patients using univariate COX analysis model and multivariate COX analysis model

Variable	Disease specific survival				Overall survival			
	Univariate analysis		Multivariate analysis		Univariate analysis		Multivariate analysis	
	HR	95% CI	HR	95% CI	HR	95% CI	HR	95% CI
Age (years)								
≤ 35	Reference		Reference		Reference		Reference	
35–55	1.485	0.784–2.811	1.387	0.728–2.641	0.796	0.541–1.171	0.789	0.535–1.165
> 55	1.352	0.695–2.630	1.327	0.675–2.609	0.768	0.509–1.160	0.837	0.549–1.275
Race								
White	Reference		Reference		Reference		Reference	
Black	1.390	0.830–2.328	0.871	0.491–1.545	1.896	1.351–2.663	1.363	0.936–1.986
Others and unknown	1.365	0.861–2.164	0.990	0.581–1.686	1.172	0.816–1.682	1.020	0.679–1.533
Laterality								
Left	Reference		Reference		Reference		Reference	
Right	0.868	0.605–1.245	0.827	0.574–1.193	0.825	0.628–1.082	0.783	0.594–1.032
Grade								
I	Reference		Reference		Reference		Reference	
II	0.490	0.251–0.958	0.981	0.451–1.868	0.412	0.228–0.754	0.670	0.361–1.246
III + IV	0.648	0.373–1.125	1.966	1.076–3.579	0.709	0.444–1.134	1.747	1.037–2.943
Unknown	0.458	0.272–0.772	1.387	0.758–2.538	0.596	0.382–0.929	1.155	0.705–1.892
Tumor size(mm)								
≤ 50	Reference		Reference		Reference		Reference	
50–100	2.828	1.747–4.579	2.373	1.425–3.953	1.901	1.361–2.654	1.758	1.216–2.541
> 100	7.238	4.606–11.374	4.494	2.590–7.796	4.111	2.969–5.694	2.921	1.938–4.404
Lymph node								
Negative	Reference		Reference		Reference		Reference	
Positive	9.746	4.741–20.035	4.159	1.735–9.966	7.712	3.935–15.114	4.152	1.912–9.019
Surgery procedure								
Mastectomy	Reference		Reference		Reference		Reference	
BCS	0.188	0.121–0.290	0.297	0.184–0.480	0.333	0.249–0.446	0.445	0.321–0.616
Radiotherapy								
No	Reference		Reference		Reference		Reference	
Yes	1.339	0.842–2.130	0.947	0.589–1.522	1.202	0.818–1.765	0.908	0.613–1.344

*DSS* disease-specific survival, *OS* overall survival, *MPTB* malignant phyllodes tumor of the breast, *HR* hazard ratio, *CI* confidence interval, *Grade* I well differentiated; II moderately differentiated; III poorly differentiated; IV undifferentiated, *BCS* breast conservation surgery

**Fig. 1:**
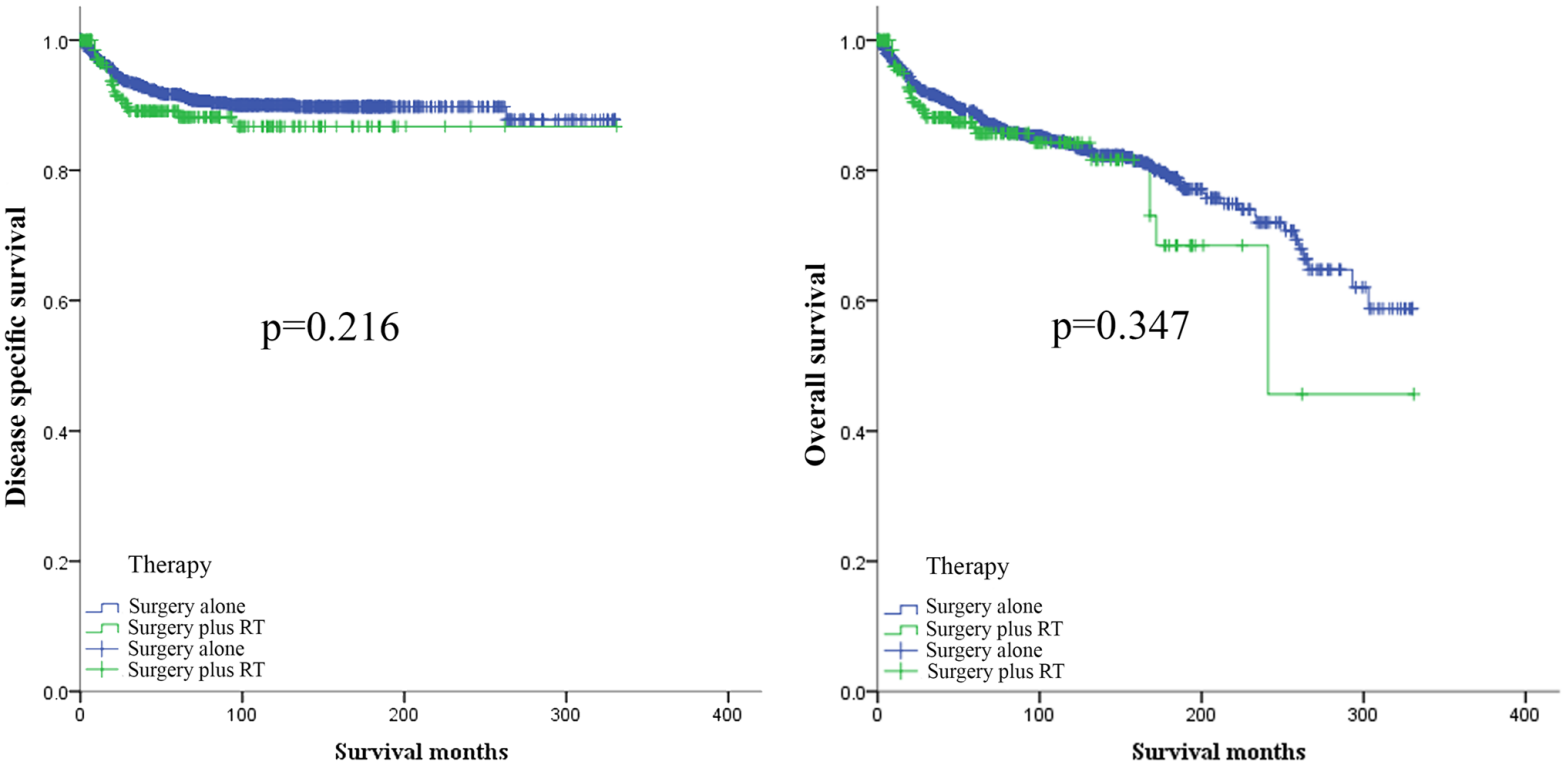
The effect of adjuvant radiotherapy in MPTB patients before PSM. *MPTB* malignant phyllodes tumor of breast, *PSM* propensity-sore matching, *RT* radiotherapy

**Fig. 2:**
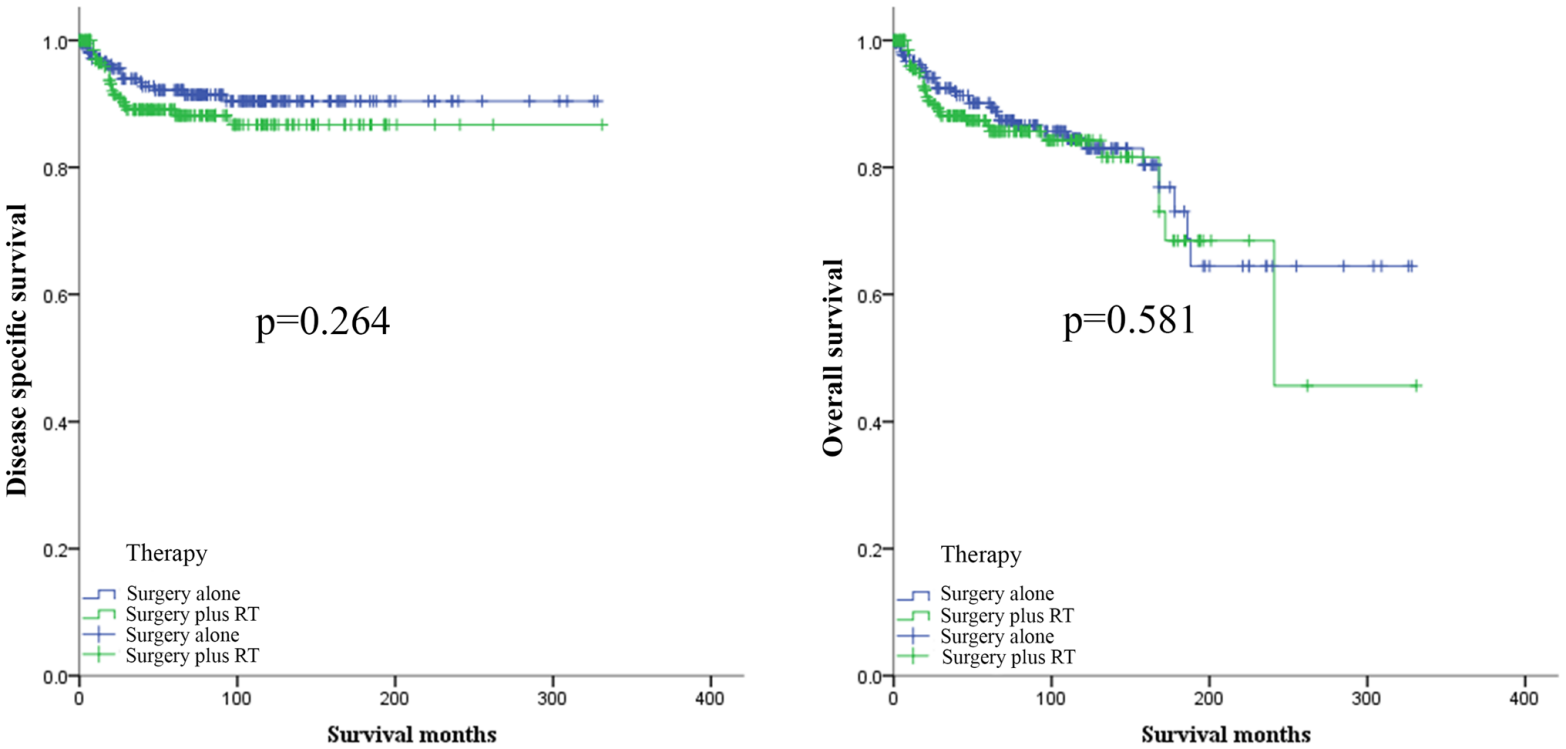
The effect of adjuvant radiotherapy in MPTB patients after PSM. *MPTB* malignant phyllodes tumor of breast, *PSM* propensity-sore matching, *RT* radiotherapy

Before PSM, Kaplan–Meier curves showed no significant difference in DSS and OS between the surgery alone cohort and the surgery plus adjuvant RT cohort (*p* = 0.264 and *p* = 0.581, respectively) (Fig. 1). To exclude the effect of clinicopathological characteristics differences between the surgery only cohort and the surgery plus adjuvant RT cohort, we used the PSM method to balance the characteristics differences. After the PSM, the characteristics of the two cohorts were balanced (Table 1). However, there was no significant difference no matter in DSS or OS (Fig. 2).

**Fig. 3:**
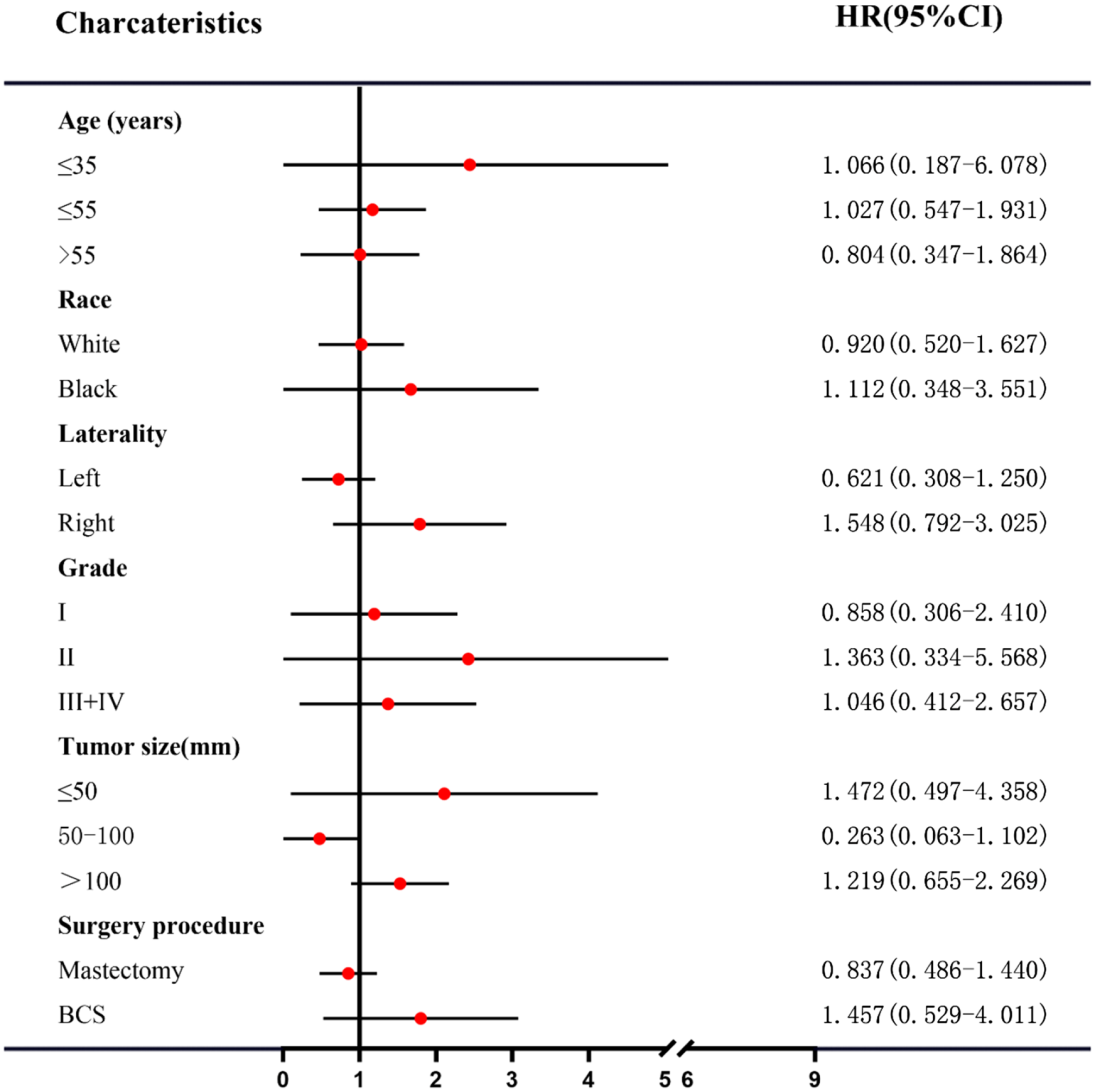
Subgroup analysis to identify the effect of adjuvant radiotherapy on BCSS of MPTB patients. *BCSS* breast cancer-specific survival, *MPTB* malignant phyllodes tumor of breast

**Fig. 4:**
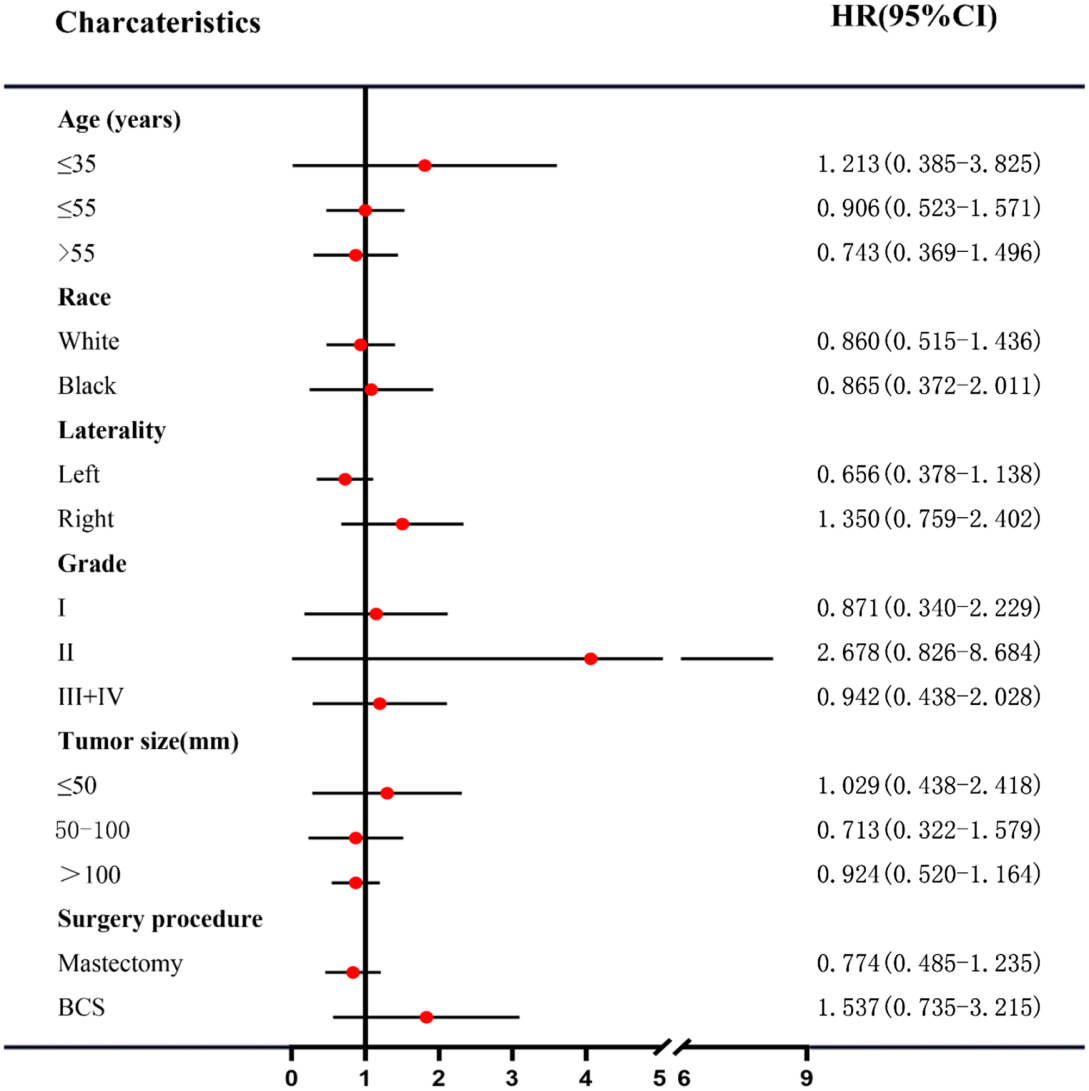
Subgroup analysis to identify the effect of adjuvant radiotherapy on OS of MPTB patients. *OS* overall survival, *MPTB* malignant phyllodes tumor of breast

We tried to identify a subgroup of MPTB patients achieving survival benefits from adjuvant RT. However, it failed and no subgroup was observed benefited from adjuvant RT (Figs. 3 and 4). As demonstrated above, tumor size was an important prognostic factor of DSS and OS, and MPTB patients with tumor of 50–100 mm or > 100 mm had worse DSS and OS (Table 2). The role of adjuvant RT in MPTB patients with tumor > 50 mm was investigated, stratified by surgery procedure. Interestingly, there was a trend that patients with tumor > 50 mm benefited from adjuvant RT if they received BCS, though it wasn’t statistically significant (Supplementary Fig. 2). However, a similar trend was not observed in those receiving mastectomy (Supplementary Fig. 3).

## Discussion

PTB is rare and the pathological diagnosis criteria are complex. According to mitotic figures, stromal cell atypia, tumor borders, stromal cell hypercellularity and overgrowth, PTB can be divided into three subtypes [3, 4], of which 35-64% are benign PTB, and the rest are divided into borderline and malignant subtype [11, 16]. It has been reported that about 500 women are diagnosed with MPTB every year in the United States. For MPTB, the tumor often showed infiltrative growth, carried with an unclear tumor border, infiltrated the surrounding tissue; stromal cells showed significant overgrowth and obvious atypia, sometimes accompanied by heterologous differentiation; mitotic activity was ≥ 10 mitotic figures/10 HPF, and bleeding and necrosis occurred in large areas. Compared with the other two subtypes, MPTB is characterized by a higher risk of local recurrence and distant metastasis [5]. The extensive pathological features of MPTB pose difficulties to the preoperative diagnosis and do not reliably predict clinical behaviors. Besides, specific parameters predictive of recurrence and survival have not been established. Having a good knowledge of the clinical characteristics in MPTB not only indicates prognosis but also helps diagnosis.

In our analysis, more than half of the patients were diagnosed at 35-55 years, consistent with previous reports [17]. About half of the MPTB patients with known differentiation grade carried tumor of poorly differentiated or undifferentiated, associated with reduced DSS and OS. Different from breast tumor of other histology significantly, 43.9% MPTB patients were diagnosed with tumors larger than 50 mm, in line with previous studies [1, 10, 11, 13, 18, 19]; and large tumor size impaired DSS and OS. Only 17 (1.3%) patients were found with lymph node metastasis in our analysis. Previous investigations also showed a low incidence of lymph node metastasis among MPTB patients [20–24]. Surgical resection is the preferred treatment. Previous studies demonstrated mastectomy cannot provide a benefit in DSS compared with BCS in MPTB patients [9, 25]. In our analysis, Kaplan–Meier survival curves showed improved survival in patients undergoing BCS than those undergoing mastectomy no matter receiving adjuvant RT or not (*p* < 0.001) (Supplementary Fig. 1), but patients receiving mastectomy tended to carry more risk factors (Supplementary 1). Besides, multivariate Cox analysis showed that BCS did improve survival. Therefore, surgeons should give priority to BCS in the context of a good cosmetic and oncologic outcome for MPTB patients.

Despite the complete surgical resection, the local failure rate is still high; and the local recurrence rate is up to 40% in all PTB patients [5, 8–10]. To control the high local recurrence, some studies have investigated the role of adjuvant therapy and most focus was put on postoperative radiation therapy. The study conducted by Gnerlich et al. was the largest analysis investigating the role of adjuvant RT on MPTB patients [14]. It included 3,120 patients with MPTB, of which 14.3% of women received adjuvant RT. They found that adjuvant RT significantly reduced local recurrence (adjusted HR 0.43, 95% CI 0.19–0.95). In other retrospective studies, local control rate of patients receiving adjuvant RT was higher when compared with those only undergoing surgery, but the characteristics between groups were uneven significantly [26, 27]. Up to now, there only existed one prospective trial. However, they treated borderline and malignant PTB patients combined, of which thirty patients (65%) had MPTB [12]. All the patients received BCS with negative margins and received adjuvant RT subsequently. All the 46 patients didn’t develop a local recurrence and it showed that margin-negative surgery combined adjuvant RT was a very effective therapy for borderline and malignant PTB in controlling local recurrence. These studies indicated that adjuvant RT improved the local control rates in MPTB patients.

Although the high local control rate achieved from adjuvant RT, whether this effect could translate into survival benefit remains controversial. The largest analysis conducted by Gnerlich et al. showed that adjuvant RT had no effect on disease-free survival or OS [14]. However, Pandey et al. found that PTB patients undergoing adjuvant RT showed improved 5-year disease-free survival compared with those who didn’t (61% vs. 25%), but it did not achieve a statistically significant difference (*p* = 0.16) [28]. Study conducted by Macdonald OK et al. included MPTB patients diagnosed at 1983-2002 registering in SEER database. They found that adjuvant RT predicted for worse DSS when implemented compared with surgery alone [9]. Another study conducted by Kim YJ and Kim K also included patients registering in the SEER database. They extended accrual period and enrolled patients diagnosed at 1983–2013. It indicated that adjuvant RT group were not inferior to the non-RT group on DSS [28]. In this retrospective analysis, the distribution of clinicopathological factors is uneven; adjuvant RT group had more adverse features such as high grade, large size, and advanced tumor extension compared with the non-RT group. In our study, we adopted the PSM method to balance the clinicopathological characteristic differences. Propensity score was estimated using the logistic regression model established from the factors potentially affecting a decision of treatment modalities, including age at diagnosis, race, laterality, differentiation grade, tumor size, lymph node metastasis and surgery procedure. Patients receiving adjuvant RT were matched to other patients based on the calculated scores with an algorithm of the nearest neighbor 1:1 matching. Table 1 showed that there existed no significant differences between the surgery plus adjuvant RT cohort and the surgery alone cohort. Survival curves showed no DSS or OS benefit from adjuvant RT among MPTB patients.

We next tried to investigate whether there existed any subgroup that would benefit from adjuvant RT. Pezner et al. included 478 PTB patients receiving treatment from 1964 to 2005 in the IMPAC National Oncology Database [29]. Five-year local control rates for patients with 0–2 cm, 2–5 cm and 5–10 cm tumors and undergoing lumpectomy were 91%, 85%, and 59%, respectively. These results indicated large tumor size associated with reduced recurrence-free survival. Besides, large tumor size was negatively correlated with DSS and OS in our analysis. BCS is an appropriate treatment for MPTB patients if a good cosmetic and oncologic outcome are feasible [9] and was identified as a protective factor. However, the large average presenting tumor size of MPTB [1, 10, 11, 13, 18] limit the surgeon’s ability to achieve negative margins with BCS alone. Considering all these factors, we next investigated the role of adjuvant RT in patients with tumor > 50 mm stratified by surgery procedure. We found that in MPTB patients with tumor > 50 mm and receiving BCS, they achieved DSS and OS benefits from adjuvant RT though there were no statistical differences. By contrast, there existed no DSS and OS benefit in patients receiving mastectomy. Our results reminded clinicians considering adjuvant RT in MPTB patients with tumor > 50 mm and receiving BCS; those receiving mastectomy may be free of postoperative radiation therapy.

We would like to acknowledge the limitations of our study. Because of the nature of retrospective analyses, we could not exclude selection bias. However, multivariate analyses and PSM method were employed to reduce potential confounding factors. Notably, some variables that play important prognostic roles among MPTB patients are not available in the SEER database, including tumor margin status, radiation dosage, and other histology factors. Finally, we failed to include local recurrence data in our study because of the absence of information in the SEER database. As one of the largest population-based analysis to date, these results indicated that adjuvant RT did not improve DSS or OS in MPTB patients.

## Electronic supplementary material

Below is the link to the electronic supplementary material.

Supplementary material 1 (DOCX 255 kb)
