# Supplementary material for: The role of adjuvant radiotherapy in patients with malignant phyllodes tumor of the breast: a propensity-score matching analysis
Source: Breast Cancer. 2020 Aug 3;28(1):110–8. doi: 10.1007/s12282-020-01135-7 (PMC7796876; doi:10.1007/s12282-020-01135-7)
Supplement: Supplementary file 1 — Supplementary material 1 (DOCX 255 kb) [file 12282_2020_1135_MOESM1_ESM.docx]

Supplementary figure 1: the effect of adjuvant radiotheapy in MPTB patients with tumor larger than 5 cm and receiving BCS.


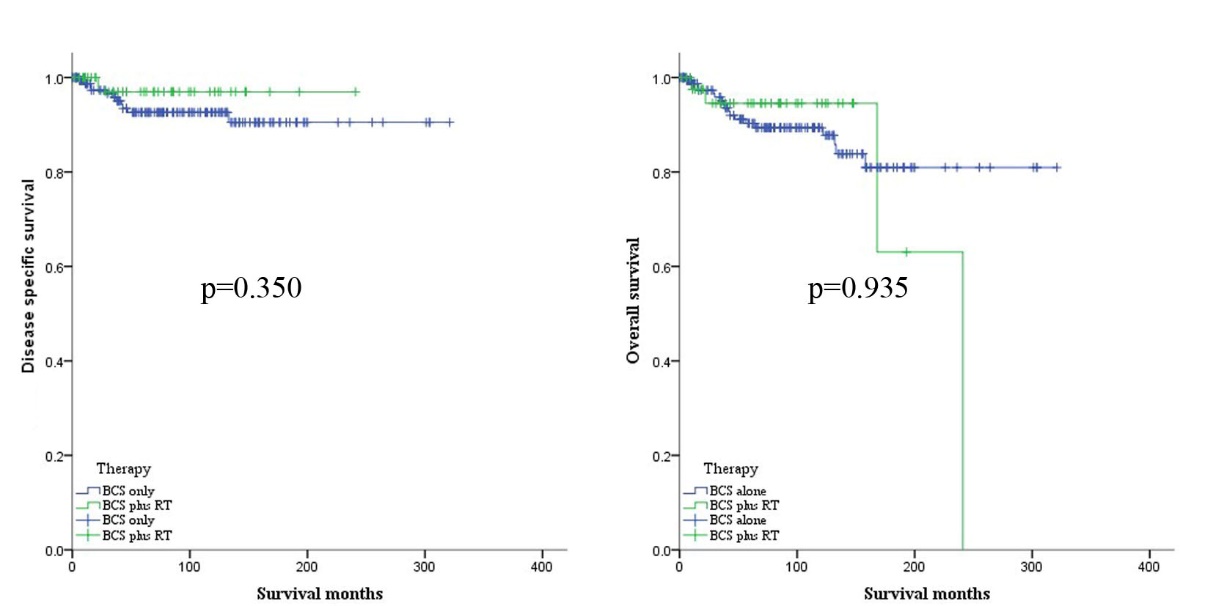


Abbreviations: MPTB: malignant phyllodes tumor of breast; BCS: breast conservation surgery; RT: radiotherapy.

Supplementary figure 2: the effect of adjuvant radiotheapy in MPTB patients with tumor larger than 5 cm and receiving mastectomy.


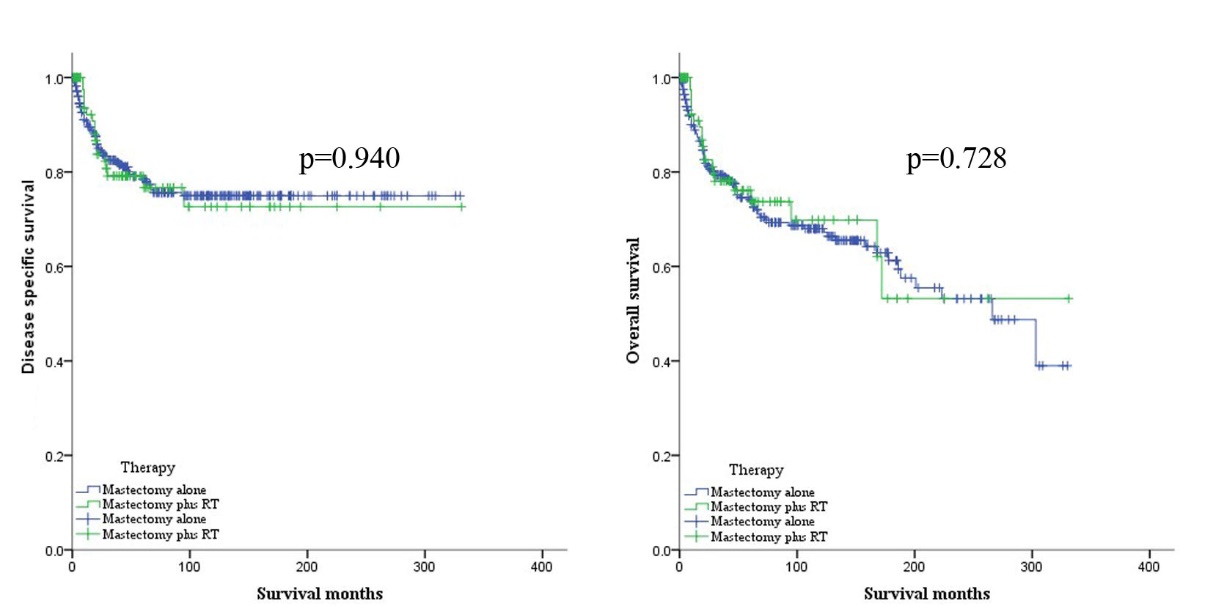


Abbreviations: MPTB: malignant phyllodes tumor of breast; RT: radiotherapy.

Supplementary figure 3: the effect of adjuvant radiotherapy in MPTB patients stratified by surgery type.


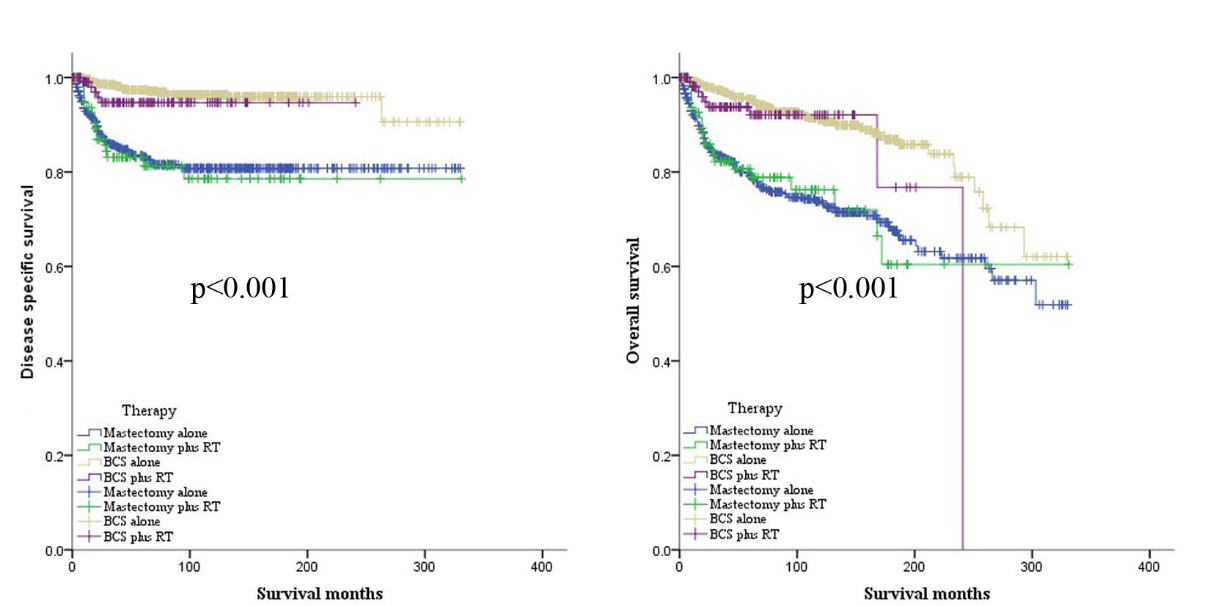


Abbreviations: MPTB: malignant phyllodes tumor of breast; RT: radiotherapy; BCS: breast conservation surgery.

Supplementary table 1: The clinicopathological characteristics of MPTB patients stratified by the surgery procedure.

|  | Mastectomy | | BCS | |  |
| --- | --- | --- | --- | --- | --- |
|  | Number | Percent | Number | Percent | p value |
| Age (years) |  |  |  |  |  |
| ≤35 | 71 | 12.6 | 106 | 13.4 | 0.129 |
| 35-55 | 309 | 54.8 | 389 | 49.3 |  |
| ＞55 | 184 | 32.6 | 294 | 37.3 |  |
| Race |  |  |  |  |  |
| White | 379 | 67.2 | 616 | 78.1 | <0.001 |
| Black | 78 | 13.8 | 72 | 9.1 |  |
| Others and unknown | 107 | 19.0 | 101 | 12.8 |  |
| Laterality |  |  |  |  |  |
| Left | 258 | 45.7 | 397 | 50.3 | 0.097 |
| Right | 306 | 54.3 | 392 | 49.7 |  |
| Grade |  |  |  |  |  |
| I | 122 | 21.6 | 40 | 5.1 | <0.001 |
| II | 90 | 16.0 | 109 | 13.8 |  |
| III or IV | 117 | 20.7 | 211 | 26.7 |  |
| Unknown | 235 | 41.7 | 429 | 54.4 |  |
| Tumor size(mm) |  |  |  |  |  |
| ≤50 | 190 | 33.7 | 569 | 72.1 | <0.001 |
| 50-100 | 179 | 31.7 | 185 | 23.4 |  |
| ＞100 | 195 | 34.6 | 35 | 4.4 |  |
| Lymph node |  |  |  |  |  |
| Negative | 552 | 97.9 | 784 | 99.4 | 0.015 |
| Positive | 12 | 2.1 | 5 | 0.6 |  |
| Radiotherapy |  |  |  |  |  |
| Yes | 111 | 19.7 | 115 | 14.6 | 0.013 |
| No | 453 | 80.3 | 674 | 85.4 |  |

Abbreviations: MPTB: malignant phyllodes tumor of the breast; BCS: breast conservation surgery; Grade: I well differentiated; II moderately differentiated; III poorly differentiated; IV undifferentiated.
